# Prevention of Lice-Borne Diseases

**Published:** 1918-10-19

**Authors:** 


### PREVENTION OF LICE-BORNE DISEASES.

### Two Disinfectors that Kill.

COLONEL WILLIAM HUNTER, C.B., A.M.S., Chief of the British Sanitary Commission to Serbia, in a lecture delivered before the Royal Society of Medicine on July 17, 1918, demonstrated convincingly the feasibility of entirely preventing the lice-borne diseases of armies in the field by very simple means. Some exercise of the imagination is required in order to realise the possible results of the elimination of such war-wasteful diseases as lice-borne typhus, relapsing and trench fevers.

Had this been accomplished early, the strength of many battalions would have accrued to the armies on the various Fronts, and an incalculable saving in hospital accommodation and in transport would have resulted. Who, also, can estimate the influence of such an addition to the fighting strength on the course of the war? Typhus and relapsing fevers have occurred only among the forces of the Balkan States and of Russia, but trench fever, bred on the Western Front, has been more universally distributed. Colonel Hunter records the introduction of an epidemic by a division of troops arriving in Salonika in December 1915.

In dealing with the great epidemics of lice-borne diseases in Serbia in 1915, the first work of the British Sanitary Mission was the initiation of two new types of lice destructors, named the "railway van disinfector" and the "Serbian barrel disinfector," respectively.

### THE RAILWAY VAN DISINFECTOR.

The former is concerned in the principle of lice destruction on the largest possible scale in the shortest period of time, by means of current steam of great volume derived from the boiler of a locomotive engine under a pressure of 4 to 7 atmospheres (temperature 144° C. to 168° C.). The van is an ordinary cattle-truck fitted with wooden racks, 3 feet wide by 3 feet deep, and with a steam-pipe 2 to 3 inches in diameter, perforated by a series of small holes, running round the floor about 2 feet from the sides. Each van can deal with 250 kits, including greatcoats and two blankets per man, every two hours.

The temperature in the centre of ten tightly-rolled blankets reaches 105° C., so that it is unnecessary to unpack the kits, they can be disinfected in bundles, duly labelled.

### COMPARATIVE WORKING CAPACITY OF THE VAN.

The working capacity in two hours' time of a double van disinfector as compared with that of the chief types used in the Army is indicated in the subjoined table.

### THE SERBIAN BARREL DISINFECTOR.

The Serbian barrel is intended for use by widely scattered and smaller groups, such as regimental companies and native villages. It consists of a wine or water barrel, or any metal tank or bin which may be procurable, through the bottom of which one large central hole is made, with a circle of 5 or 6 small holes around it. A wooden cover is provided, and a perforated diaphragm about 9 inches off the bottom, in order to keep the clothes away from the steam-holes. It stands on a circular boiler, a narrow sausage ring filled with sand intervening, in order to prevent the loss of steam. A hole dug in the ground suffices for the fireplace.

One barrel disinfector to each company is recommended; it is capable of disinfecting the kits of a company every two or three weeks. The time required for disinfection is one hour from the raising of steam.

Evidence of the success of this system of disinfection is conclusive. The double epidemic of typhus and relapsing fever commenced in January and reached its climax in March, when preventive measures were started which were followed in a fortnight by a fall which was maintained at a uniform rate up to June, when the epidemic was arrested.

The world is indebted to Lieut.-Colonel G. F. Stammers, R.A.M.C., of the British Serbian Sanitary Commission, for an idea born of emergency, the adaptation of which to the armies on the Western Front should lead to incalculable results.

The railway vans and engines, and the wine barrels, requisitioned in an emergency, are unsuitable for an organised Sanitary Service—the Sanitary Sections of the R.A.M.C. struggled valiantly to make the latter a success on the Western Front, but owing to local conditions it was impossible to maintain their supply and organisation. It is doubtful whether railway facilities, either of rolling stock or of engines, would be available, as the demands of supply are so urgent; possibly some of the small engines of the trench railways might be available during the hours of daylight.

The requirements would be met more suitably by the creation of a steam Motor Convoy of disinfecting cars, organised and administered on lines similar to those of the Motor Ambulance Convoys.

They should be of about 300 cubic feet capacity and provided with high-pressure boilers which would supply steam for locomotion and for disinfection. The pressure in the boilers should be sufficient to raise the temperature in the cars to a degree capable of vaporising the water of condensa-

CAPACITY OF VARIOUS TYPES.

| | No. of Loads | No. of Blankets | No. of Kits |
|---|---|---|---|
| 1. Thresh, portable ... ... | 2 | 60 | 40* |
| 2. Thresh on Foden lorry (two chambers) ... ... | 2 | 240 | 140* |
| 3. Lyon, portable, M.O. ... | 2 | 120 | 70* |
| 4. Lyon, fixed, L.O. ... ... | 2 | 260 | 120* |
| 5. Lyon, No. 2 D ... ... | 2 | 120 | 70* |
| 6. Double van disinfector ... | 1 | 1,500 | 500† |

* Less overcoats and blankets.
† Plus 500 overcoats and 1,000 blankets.

**Prevention of Lice-Borne Diseases—(*continued*).**

tion in order that the disinfecting process may be a dry one. The tubular boilers used in fire engines would be the most suitable type; they are light in weight, of high pressure, and rapid in action. Such a car would be capable of disinfecting sixty to seventy complete kits per hour, including greatcoats and blankets, so that one per 1,000 troops, or 120 per army, would be sufficient. These Motor Disinfecting Convoys, placed under the control of an Army Headquarters, could be distributed to meet the requirements of the various formations in a manner similar to that common to the Motor Ambulance Convoys and Sanitary Sections.

The disinfection of the greatest number in the shortest period is not necessary under ordinary circumstances. The essential principle to be observed in the delousing of armies is disinfection by groups, the simultaneous disinfection of the bedding, clothing, and occupants of sleeping quarters used in common; it is useless to disinfect those off duty, and to lodge the still infested duty man with them, for in one night the whole are reinfected.

A very large proportion of the wastage of man-power on the Western Front is attributable to the only partially contested ravages of lice, acari, and streptococci. With a thorough system of disinfection, trench fever, scabies, and impetigo would disappear; there would result a corresponding increase in fighting strength, a reduction of hospital beds, and a welcome relief to strained doctors and nurses.

The present arrangements for the disinfection of underclothing and for its distribution at the baths are satisfactory, but jackets, trousers, waistcoats, blankets, and greatcoats require equal consideration. The hot-ironing of trouser seams, the isolated use of improved disinfectors by enthusiasts, and the occasional piecemeal disinfection of blankets can have but a questionable influence on the situation. Enormous strides in disinfection have been made in the Army during the war. The latest disinfectors, the twin "Thresh" on "Foden" lorries, are very capable machines; their performances, however, do not equal those published by Colonel Hunter, they are very costly, and unobtainable in sufficient numbers. They would, however, form a useful nucleus for the proposed convoy system.

That ailments preventable by disinfection still hold up considerable forces is a matter for deep concern; it is a subject that is not realised either by the Army or by the public, because they are not instructed. The wide publication of the war wastage due to these causes, and the means of their prevention, would surely lead to a trial of the system which proved so successful in the stamping out of lice-borne diseases in Serbia.

